# Multisensory and modality specific processing of visual speech in different regions of the premotor cortex

**DOI:** 10.3389/fpsyg.2014.00389

**Published:** 2014-05-05

**Authors:** Daniel E. Callan, Jeffery A. Jones, Akiko Callan

**Affiliations:** ^1^Center for Information and Neural Networks, National Institute of Information and Communications Technology, Osaka UniversityOsaka, Japan; ^2^Multisensory Cognition and Computation Laboratory Universal Communication Research Institute, National Institute of Information and Communications TechnologyKyoto, Japan; ^3^Psychology Department, Laurier Centre for Cognitive Neuroscience, Wilfrid Laurier University, WaterlooON, Canada

**Keywords:** audio-visual, premotor, multisensory, mirror system, fMRI, internal model

## Abstract

Behavioral and neuroimaging studies have demonstrated that brain regions involved with speech production also support speech perception, especially under degraded conditions. The premotor cortex (PMC) has been shown to be active during both observation and execution of action (“Mirror System” properties), and may facilitate speech perception by mapping unimodal and multimodal sensory features onto articulatory speech gestures. For this functional magnetic resonance imaging (fMRI) study, participants identified vowels produced by a speaker in audio-visual (saw the speaker's articulating face and heard her voice), visual only (only saw the speaker's articulating face), and audio only (only heard the speaker's voice) conditions with varying audio signal-to-noise ratios in order to determine the regions of the PMC involved with multisensory and modality specific processing of visual speech gestures. The task was designed so that identification could be made with a high level of accuracy from visual only stimuli to control for task difficulty and differences in intelligibility. The results of the functional magnetic resonance imaging (fMRI) analysis for visual only and audio-visual conditions showed overlapping activity in inferior frontal gyrus and PMC. The left ventral inferior premotor cortex (PMvi) showed properties of multimodal (audio-visual) enhancement with a degraded auditory signal. The left inferior parietal lobule and right cerebellum also showed these properties. The left ventral superior and dorsal premotor cortex (PMvs/PMd) did not show this multisensory enhancement effect, but there was greater activity for the visual only over audio-visual conditions in these areas. The results suggest that the inferior regions of the ventral premotor cortex are involved with integrating multisensory information, whereas, more superior and dorsal regions of the PMC are involved with mapping unimodal (in this case visual) sensory features of the speech signal with articulatory speech gestures.

## Introduction

Visual observation of gestural information available from a speaker's face improves speech perception, especially under noisy conditions (Sumby and Pollack, 1954; Grant and Braida, 1991; Callan et al., 2001, 2003). Speech gesture information, which consists of the biological motion of the various articulators (jaw, lips, tongue, larynx) that specify vocal tract shape, facilitates speech perception because of the direct relationship between vocal tract shape, speech acoustics, and the dynamic deformation of the skin of the face. Brain imaging studies suggest that the brain regions involved in the integration of multisensory information process gestural speech information to facilitate speech perception (Callan et al., 2003, 2004a,b; Skipper et al., 2007a,b). One means by which speech intelligibility may be enhanced by the addition of visual information is via brain regions that are involved in the multisensory integration process. Integration of temporally concordant information from multiple sensory channels (e.g., auditory and visual modalities) within specific brain regions, such as the superior temporal gyrus/sulcus (STG/S) in the case of audio-visual speech (Calvert et al., 2000; Callan et al., 2001, 2003; Sekiyama et al., 2003), results in enhanced neural activity that is greater than the combined activity in response to unimodal speech stimuli presented alone.

Another property of multisensory integration is the principle of inverse effectiveness, which asserts that multisensory enhancement is greatest under conditions in which unimodal stimuli elicit weak neural responses (e.g., due to subthreshold stimulation, noisy conditions; Wallace et al., 1992; Stein and Meredith, 1993). This multisensory enhancement effectively increases perceptual acuity and is maximized by temporally and spatially concordant stimulation of different sensory modalities (e.g., auditory and visual) (Stein and Meredith, 1993). The STG/S as well as the inferior frontal gyrus IFG/Broca's area have been shown to be involved in multisensory enhancement during perception of audio-visual speech in noise (Callan et al., 2001, 2003, 2004b; Alho et al., 2012).

Many researchers have proposed that speech intelligibility is enhanced by visual speech cues because the information available in the visible gestures activates motor representations that can be used to constrain auditory speech perception. Specifically, researchers hypothesize that certain brain regions internally model and simulate speech production and that these internal models are used to recover vocal tract shape information inherent in the speech signal (Callan et al., 2003, 2004a; Wilson and Iacoboni, 2006; Iacoboni and Wilson, 2006; Skipper et al., 2007a,b; Iacoboni, 2008; Poeppel et al., 2008; Rauschecker and Scott, 2009; Rauschecker, 2011). Internal models are a well-known concept in the motor control literature, and are believed to be used by the brain to simulate the input/output characteristics, or their inverses, of the motor control system (Kawato, 1999). In the case of speech, the forward and inverse mappings of the relationship between aspects of speech articulation and the acoustic features of speech output (as well as the orosensory and visual properties of speech) may be used to facilitate speech perception. Forward internal models predict the sensory (auditory, orosensory) consequences of the actions of speech articulation, whereas, inverse internal models determine the motor commands needed to articulate a desired sensory (auditory, orosensory) target. Callan et al. (2004a, 2010) suggested that the auditory consequences of internally simulated articulatory control signals (articulatory-auditory internal models for various phonemes) are used to constrain and facilitate speech perception under ambiguous conditions (e.g., speech perception in noisy environments, or the perception of non-native speech) through the competitive selection of the internal model that best matches the ongoing auditory signal. These internal models are thought to be instantiated in a network of speech motor regions that include the PMC and Broca's area, auditory processing regions STG/S, the IPL, and the cerebellum. Other researchers such as Rauschecker and Scott (2009) have discussed the use of forward and inverse auditory—articulatory mappings (utilizing principles of internal models) for speech perception and production, and have suggested that the IPL serves as an interface for matching of these mappings.

Several theories have proposed that speech perception uses aspects of speech production to extract phonetic information from sensory stimulation: Motor theory (Liberman et al., 1967), revised motor theory (Liberman and Mattingly, 1985; Liberman and Whalen, 2000), and various constructivist based theories (Callan et al., 2004a, 2010; Skipper et al., 2007a; Rauschecker and Scott, 2009; Rauschecker, 2011) including the Perception for Action Control Theory (PACT) (Schwartz et al., 2012). The observation of Mirror Neuron system like properties (active both during observation and execution of action) in Broca's area, the ventral inferior premotor cortex (PMvi) and the ventral superior and dorsal premotor cortex (PMvs/PMd), during speech production and perception has provided support for theories that propose a role for the motor system in speech perception (Callan et al., 2000a,b, 2006a,b, 2010; Wilson et al., 2004; Nishitani et al., 2005; Meister et al., 2007).

A number of studies have shown that these brain regions that appear to have Mirror Neuron system like properties, such as Broca's area and premotor cortex (PMC), respond to audio, visual, and audio-visual speech information (Campbell et al., 2001; Bernstein et al., 2002; Nishitani and Hari, 2002; Olson et al., 2002; Callan et al., 2003, 2004a,b; Paulesu et al., 2003; Calvert and Campbell, 2003; Ojanen et al., 2005; Skipper et al., 2005, 2007b; Alho et al., 2012; Dubois et al., 2012; Mashal et al., 2012). As well, the cerebellum has been shown to be involved in both perception and production of speech and is thought to instantiate processes related to internal models (Kawato, 1999; Imamizu et al., 2000; Callan et al., 2004a, 2007; Rauschecker, 2011; Tourville and Guenther, 2011; Callan and Manto, 2013). The objective of this study is to determine if these various brain regions (Broca's area, PMC, and the cerebellum) differentially process visual speech information, in the context of multisensory integration as well as during modality specific extraction of features to recover speech gesture information.

One potential confound that may exist for many studies that have investigated the brain regions involved with processing visual speech gesture information is the inability to distinguish whether the brain activity reflected processing of the visual gestural speech information or whether the brain activity reflected improved intelligibility that resulted from processes carried out elsewhere. Activity observed in many of the same brain regions thought to be involved with facilitative processing of visual speech information, including the PMC, Broca's area, Sylvian parietal temporal area Spt, IPL, and STG/S, have also been shown to be involved in increased intelligibility and comprehension (Callan et al., 2010; Londei et al., 2010). For studies of audio-visual speech processing this confound exists because in many cases the addition of visual speech gesture information improves intelligibility. A related confound is that it is often the case that these same brain regions (IFG, PMC, and cerebellum) involved with speech processing are also activated when task demands are high and require more working memory and attention (Jonides et al., 1998; Davachi et al., 2001; Sato et al., 2009; Alho et al., 2012). The activation of these regions may be related to task difficulty, greater attentional demand, and working memory (including internal rehearsal) that may be independent from specific processes involved with mapping between articulatory and auditory representations for speech perception. This increase in task demands occurs for most visual only speech tasks as well as for speech in noise tasks.

In this study the task was designed to control for both intelligibility and task difficulty by ensuring that performance using visual information alone was the same as that under the audio-visual conditions of interest. Specifically, we asked participants to identify vowels in visual and audio-visual speech stimuli. For this task, the visual information alone allowed for very high perceptual performance. Analyses focused on two regions of the PMC and the cerebellum, which have been previously shown to have mirror system properties and are thought to be involved in the instantiation of internal models (Callan et al., 2000a, 2004a, 2006a,b, 2010; Wilson et al., 2004; Skipper et al., 2007a). These regions are active during processing of visual speech information (Campbell et al., 2001; Bernstein et al., 2002; Nishitani and Hari, 2002; Olson et al., 2002; Callan et al., 2003, 2004a,b; Calvert and Campbell, 2003; Paulesu et al., 2003; Ojanen et al., 2005; Saito et al., 2005; Skipper et al., 2005, 2007b; Alho et al., 2012; Dubois et al., 2012; Mashal et al., 2012). One of these regions in the PMC is more inferior and includes Broca's area and the PMvi. The other region is more superior and/or dorsal and has been referred to as PMvs and PMd.

It is rather uncontroversial that during the development of speech production, auditory-articulatory and orosensory-articulatory relationships must be established and encoded into internal models (Callan et al., 2000b; Tourville and Guenther, 2011; Guenther and Vladusich, 2012). Acoustic and orosensory signals are direct products of one's own articulation at are one goal of speech production. Likewise, internal models for visual aspects of speech (visual-auditory and visual-articulatory mappings) are learned by mapping features of speech gestures in the visual speech signal to the corresponding acoustics as well as to the articulations necessary to produce the corresponding deformation of the face. A primary goal of this study is to determine if the brain regions thought to instantiate internal models for speech (Broca's/PMvi, PMvs/PMd, IPL, Cerebellum) differ in their processing of audio-visual and visual only speech with respect to multisensory integration and modality specific extraction of articulatory speech gesture information (unimodal features in stimulation that specify phonemes). To accomplish this goal we identified the brain activity present during audio-visual and visual only speech processing. Given the results of previous experiments we hypothesized that both the PMvi/Broca's and PMvs/PMd would be active in both conditions. We further hypothesized the PMvi/Broca's area to be a site in which auditory and articulatory gesture information converge, and therefore activation in this area would show properties of multisensory enhancement. In contrast, a more prominent role for the PMvs/PMd may be the processing of modality specific speech gesture information. To determine which brain regions would show properties of multisensory enhancement we investigated differences in brain activity between audio-visual and audio only conditions at different signal-to-noise ratios. Based on the principle of inverse effectiveness (Wallace et al., 1992; Stein and Meredith, 1993) it was hypothesized that multisensory enhancement regions would show greater activity when unimodal audio stimuli had a lower signal-to-noise ratio.

## Methods

### Subjects

Sixteen 21–43 year-old (6 women and 10 men) right-handed subjects participated in this study. Eight subjects spoke English as their first language. The other eight subjects were native Japanese speakers who were proficient English speakers. The Japanese speakers all learned English beginning at 13 years of age or younger, and use English as their primary language at work and socially. Subjects gave written informed consent. The experimental procedures were approved by the ATR Human Subject Review Committee and were carried out in accordance with the principles expressed in the WMA Declaration of Helsinki.

### Procedure

#### Conditions

The experiment consisted of 10 conditions, however, only eight conditions were analyzed for this study. These eight conditions included: (1) an audiovisual condition (AV) where subjects saw a movie of the face articulating speech and heard the speaker utter a consonant-vowel-consonant (CVC) English monoslyllabic word with background audio noise (multispeaker babble) presented at three signal-to-noise ratios (−6, −10, and −14 dB; referred to as conditions AV6, AV10, AV14, respectively); (2) an audio only condition (A) where subjects saw a still face image while listening to the CVC with background audio noise at the same three signal-to-noise ratios (−6, −10, and −14 dB; referred to as conditions A6, A10, A14, respectively); (3) a visual only condition (VO) where subjects saw a movie of the face articulating speech, but without hearing the corresponding audio speech information or the audio noise; (4) and a baseline still face condition where subjects saw a still face but heard no audio. It should be note that in the same fMRI session subjects saw a still face with audio noise (SN) and a visual only condition with audio noise (VN) for a different study. The sound pressure level for the auditory stimuli was approximately 85–90 dB SPL. The stimuli were constructed such that the random segments of multispeaker babble noise were kept at a constant level and the speech signals were added to the babble noise at the specific signal to noise ratios (−6, −10, and −14 dB).

#### Protocol

The experiment consisted of a two-alternative forced choice task in which subjects identified by button press with their left thumb which vowel was present in the CVC English monoslyllabic word presented. In the baseline still face condition the subject randomly pushed one of the two buttons. The speech stimuli were spoken by a female native English speaker. Each presentation was 1 s in duration for all trials. For trials with visual speech this 1-s included facial motion before and after the audio speech signal for the word. The trial lasted approximately 3.9 s with ±200 ms of random jitter. The audio noise mixed with the speech signal consisted of an English multispeaker babble track (Audiotec, St. Louis, MO, USA). Multispeaker babble is known to be an effective and central masker of speech as its main energy is in the same range as the word stimuli (Wilson and Strouse, 2002). Three different runs were conducted each consisting of a separate vowel pair to be identified. The different vowel pairs consisted of /o-e/, /o-i/, and /o-^∧^/ (^∧^ as in gun). The stimuli were all common English words with pairs containing the same consonants (see Table 1 for the list of stimuli). The left or right position of the button press for the /o/ response was counterbalanced across subjects and remained the same throughout the experiment for a single subject. Subjects were given practice trials before the experiment so they were familiar with the task and button response positions. Subjects were instructed to press the button to identify the vowel after presentation of each 1-s stimuli. The experimenter verbally instructed the subjects which button position was associated with each vowel before each run. There were seven different word pair stimuli for each vowel contrast (14 words for each vowel contrast). The same words were used for all the AV, A, and VO conditions. A blocked presentation design was implemented in which seven trials of the same condition were presented in succession for one block. The order of presentation of the various conditions was randomized. Subjects underwent three runs of fMRI scanning. Each run corresponded to a different vowel contrast to be identified, /o-e/, /o-i/, and /o-^∧^/. The order of the vowel contrast runs was randomized across subjects. There were 20 blocks in each run. Each block lasted approximately 27.5 s. The 10 conditions were randomly presented in blocks of seven trials twice during each run. A block of seven trials for each condition was presented once before a block of trials of the same condition was presented the second time. In total there were 140 trials per run.

**Table 1:** **Stimulus word pairs used in experiment**.

**/o/-/e/**	**/o/-/i/**	**/o/-/^∧^/**
Cope–cape	Boat–beat	Coat–cut
Foam–fame	Gross–grease	Dome–dumb
Grove–grave	Load–lead	Phone–fun
Post–paste	Note–neat	Mode–mud
Prose–praise	Slope–sleep	Most–must
Toast–taste	Spoke–speak	Roast–rust
Woke–wake	Those–these	Tone–ton

### fMRI data collection and preprocessing

The visual speech signal was presented by means of a computer with specialized hardware and software that interfaced with a laser disk player containing the stimuli. The laser disk player was connected to the video projector. The video from the projector located outside of the MR room was directed to a mirror positioned inside of the head coil just above the subjects' eyes. The audio was presented via a sound file on the computer (pre-mixed based on SNR) via MR-compatible headphones (Hitachi Advanced Systems' ceramic transducer headphones). The presentation of visual and audio signals using the computer hardware that controlled the laser disk ensured that there was no audio-visual asynchrony.

Brain imaging was conducted using a Shimadzu-Marconi's Magnex Eclipse 1.5T PD250 at the ATR Brain Activity Imaging Center. Functional T2^*^ weighted images were acquired using a gradient echoplanar imaging sequence (*TR* = 3.93 s). An interleaved sequence was used consisting of 37 axial slices with a 4 × 4 × 4 mm voxel resolution covering the cortex and cerebellum. Isotropic voxels were used to avoid possible distortion in realignment and normalization that occur with anisotropic voxels. For the scanner used in this study 3 mm voxels would have resulted in a longer than desired TR for each scan. Each run consisted of 140 scans. Images were preprocessed using programs within SPM8 (Wellcome Department of Cognitive Neurology, UCL). Differences in acquisition time between slices were accounted for, images were realigned and spatially normalized to MNI space (3 × 3 × 3 mm voxels) using the SPM template EPI image, and were smoothed using a 8 × 8 × 8 mm FWHM Gaussian kernel. Regional brain activity for the various conditions was assessed using a general linear model employing a boxcar function convolved with a hemodynamic response function (global normalization and grand mean scaling were used to reduce artifacts). The baseline still face condition was implicitly modeled in the design. The nine other conditions were included in the SPM model. A fixed-effect analysis was first employed for all contrasts of interest for each subject. The contrast estimates of this analysis for each subject were used for random effects analysis. The contrasts of interest included the following: VO, AV (Combined Conditions AV6, AV10, AV14), VO-AV, AV-VO, multisensory enhancement (AV10-A10)-(AV6-A6) and (AV14-A14)-(AV10-A10). The threshold for significance was set at *p* < 0.05 using a False Discovery Rate FDR correction for multiple comparisons across the entire volume using a spatial extent threshold of 20 voxels. If no voxels were found to be significant using the FDR correction a threshold of *p* < 0.001 uncorrected with a spatial extent threshold of 20 voxels was used. Region of interest analyses were conducted using MNI coordinates for the PMv/IFG (−54, 6, 12), PMvs (−48, 0, 51), and the cerebellum (−12, −72, −45; 12, −72, −45) given in Callan et al. (2003) that were found to be important for audio visual processing. Bilateral coordinates in the cerebellum were used because studies have reported activity in both the left and right cerebellum in response to audio-visual speech (Callan et al., 2003; Saito et al., 2005; Skipper et al., 2005). Additionally, it is known that the cerebellum has predominantly crossed connections to the cortex such that the right hemisphere of the cerebellum projects to the language dominant left frontal areas including the PMC (Middleton and Strick, 1997; Schmahmann and Pandya, 1997). Small volume correction for multiple comparisons (pFWE < 0.05) were carried out using the seed voxels reported above within a sphere with a radius of 10 mm.

## Results

### Behavioral results

#### Conditions showing better than chance performance

**Figure 1:**
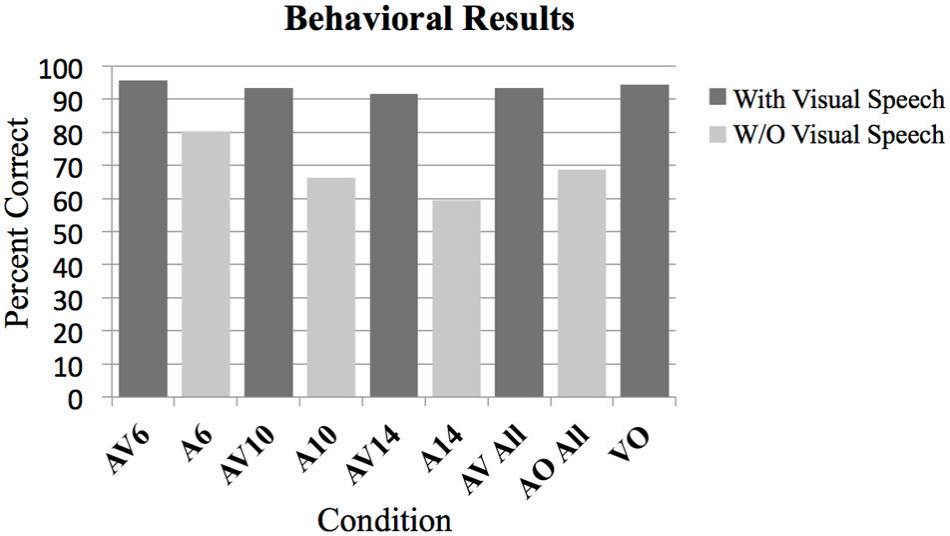
**Behavioral results as measured by percent correct on the two-alternative forced choice vowel identification task for the following conditions**. Audio Visual AV6 (−6 dB SNR), Audio A6 (−6 dB SNR), AV10, A10, AV14, A14, All AV conditions combined, all A conditions combined, Video with noise VN, and Video only VO without noise. All contrasts were significantly greater than chance performance of 50% (*p* < 0.01).

*T*-tests were used to determine which conditions showed performance that was significantly above chance on the two-alternative forced-choice vowel identification task (chance = 50%). There were 9 comparisons made altogether including the following: AV6, A6, AV10, A10, AV14, A14, AV All, A All, and VO. Bonferroni corrections for multiple comparisons were used to determine statistical significance at *p* < 0.05. Results of the analyses are presented in Figure 1 and Table 2.

**Table 2:** ***T*-Tests for conditions evaluating better than chance performance**.

**Condition**	**Mean %**	***SE* %**	***T***	**Correct *p***
AV6	95.6	1.1	43.3	*p* < 0.05^*^
A6	80.3	2.9	10.4	*p* < 0.05^*^
AV10	93.4	2.0	21.7	*p* < 0.05^*^
A10	66.3	2.6	5.9	*p* < 0.05^*^
AV14	91.6	1.5	28.0	*p* < 0.05^*^
A14	59.5	2.7	3.5	p > 0.05
AV All	93.5	1.2	34.9	*p* < 0.05^*^
A All	68.7	2.3	8.0	*p* < 0.05^*^
VO	94.4	1.2	37.9	*p* < 0.05^*^

Chance Performance was 50%. AV6, Audio-Visual −6 dB signal-to-noise ratio; A6 Audio Only −6 dB; AV10, Audio-Visual −10 dB; A10 Audio Only −10 dB; AV14, Audio-Visual −14 dB; A14 Audio Only −14 dB; SE, Standard Error;

* *significant using the Bonferroni correction for multiple comparisons*.

#### Audio-visual greater than audio only

**Figure 2:**
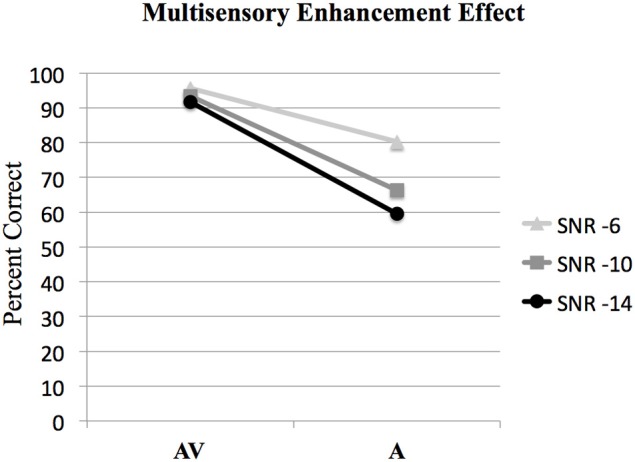
**Behavioral results showing the interaction of audio-visual enhancement at each of the signal-to-noise ratios SNRs**. The interaction of (AV6-A6)-(AV10-A10) was statistically significant [*F*_(1, 15)_ = 12.6, *p* < 0.005]; however the interaction of (AV10-A10)-(AV14-A14) was not significant [*F*_(1, 15)_ = 3.9, *p* > 0.05].

A Two-Way analysis of Variance ANOVA was conducted over factors of Modality (with levels audio-visual and audio only) and SNR (with levels −6, −10, and −14 dB). Bonferroni corrections for multiple comparisons were used to determine statistical significance at *p* < 0.05 for planned ANOVA interaction and pairwise comparison analyses. In total there were seven planned analyses. The omnibus ANOVA indicated significant interaction between Modality and SNR, *F*_(2, 95)_ = 7.1, *p* < 0.05; and significant main effects of Modality (AV > A), *F*_(1, 95)_ = 179.2, *p* < 0.05, and SNR, *F*_(2, 95)_ = 15.49, *p* < 0.05. Planned pairwise comparisons (corrected for multiple comparisons) indicated statistically significant differences between the AV conditions and the A conditions (AV6-A6: *T* = 5.79, *p* < 0.05; AV10-A10: *T* = 14.13, *p* < 0.05, AV14-A14: *T* = 14.2, *p* < 0.05; AV > A: *T* = 18.5, *p* < 0.05; AV not significantly different from VO: *T* = 0.69; see Figures 1, 2). The planned interaction analyses are given below.

#### Multisensory enhancement effect

ANOVA was used to investigate interactions between AV and A conditions at different SNR levels to determine the presence of the multisensory enhancement effect. Bonferroni corrections for multiple comparisons were used to determine statistical significance at *p* < 0.05 for all analyses. The results of the analysis of the interaction between audio and visual conditions denoting the audio-visual enhancement effect are given in Figure 2. The interaction of (AV6-A6)-(AV10-A10) was statistically significant, [*F*_(1, 63)_ = 8.2, *p* < 0.05]. However, the interaction of (AV10-A10)-(AV14-A14) was not significant, *F*_(1, 63)_ = 1.4, *p* > 0.05 (see Figure 2).

#### Controlling for performance for conditions containing visual information

One of the goals of this experiment was to control for intelligibility and task difficulty across the different conditions containing visual information to determine which brain regions are involved with multisensory and visual speech gesture information processing. No significant difference was found between the combined audio-visual conditions AV and the VO condition using a lenient uncorrected threshold (*T* = 0.69, *p* > 0.1). This null effect is important for interpreting the fMRI results because ensuring that the perceptual performance across the conditions containing visual information did not differ was necessary (see Figure 1).

### Brain imaging results

**Figure 3:**
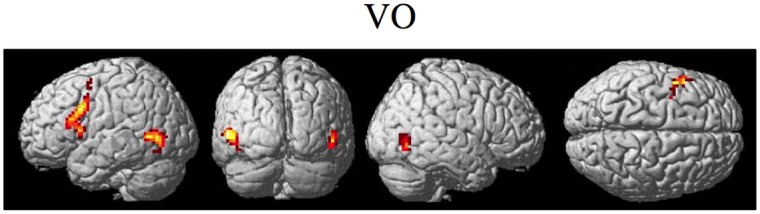
**Significant brain activity for the VO condition thresholded at pFDR < 0.05 corrected**. Activity was present in the left PMvi/Broca's, left PMvs/PMd, and left and right MT/V5 visual motion processing area.

**Figure 4:**
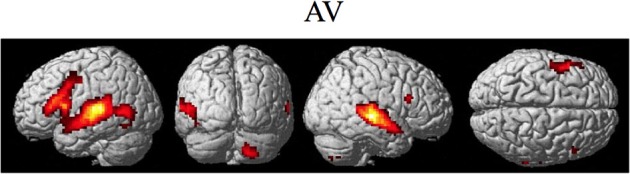
**Significant brain activity for the combined AV conditions thresholded at pFDR < 0.05 corrected**. Activity was present in left and right PMvi/Broca's area, left PMvs/PMd, left and right STG/S including primary and secondary auditory cortex, left MT/V5 visual motion processing area, and the right cerebellum lobule VIIb.

**Figure 5:**
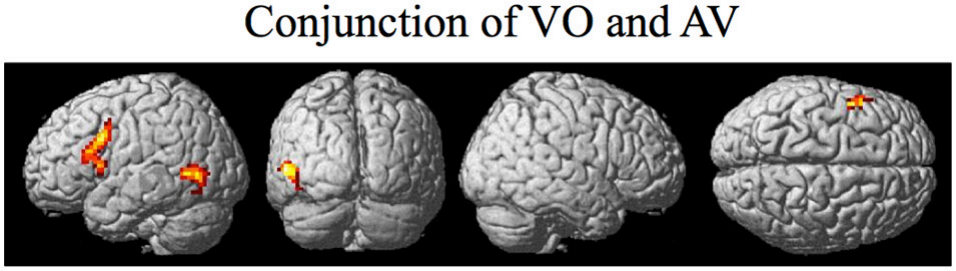
**Brain activity that was significant for both (conjunction) the VO and the combined AV conditions thresholded at pFDR < 0.05 corrected**. Activity was present in the left PMvi/Broca's, left PMvs/PMd, and left MT/V5 visual motion processing area.

**Figure 6:**
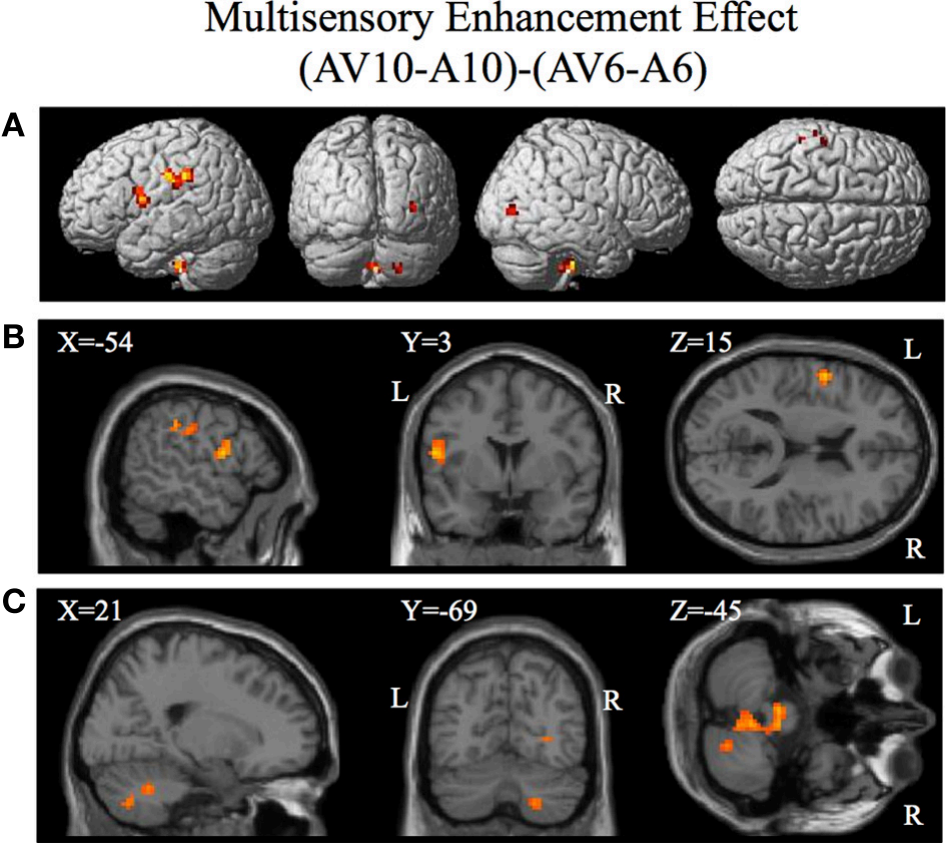
**Significant brain activity for the contrast that investigated the multisensory enhancement effect (AV10-A10)-(AV6-A6) thresholded at *p* < 0.001 uncorrected**. Activity was present in left PMvi/Broca's area, left pre- and post-central gyrus, left inferior parietal cortex and suprmarginal gyrus, the right occipital lobe, the right cerebellum lobule VIIb and IX, and the left and right brain stem. **(A)** Activity rendered on the surface of the left, back, right, and top of the brain. **(B)** Section through brain taken at MNI coordinate −54, 3, 15 shows activity that was present in the PMvi and Broca's region. **(C)** Section through brain taken at MNI coordinate 21, −69, −45 shows activity that was present in cerebellum lobule VIIb. L, left side of brain; R, right side of brain.

**Figure 7:**
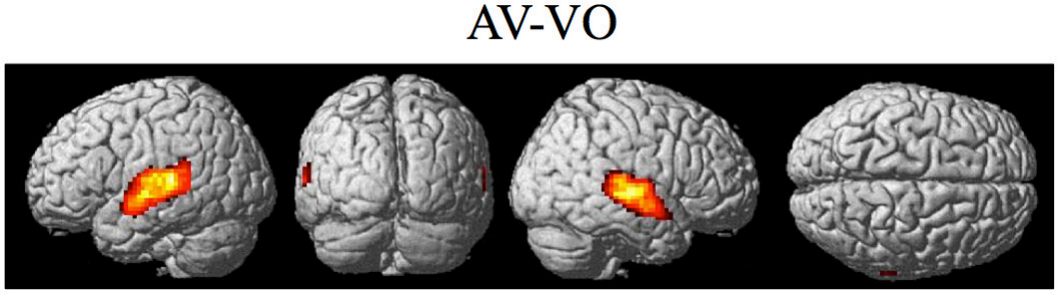
**Significant brain activity for the contrast of the combined AV conditions relative to the visual only VO condition thresholded at pFDR < 0.05 corrected**. Activity was present in the left and right superior temporal gyrus/sulcus including primary and secondary auditory cortex.

**Figure 8:**
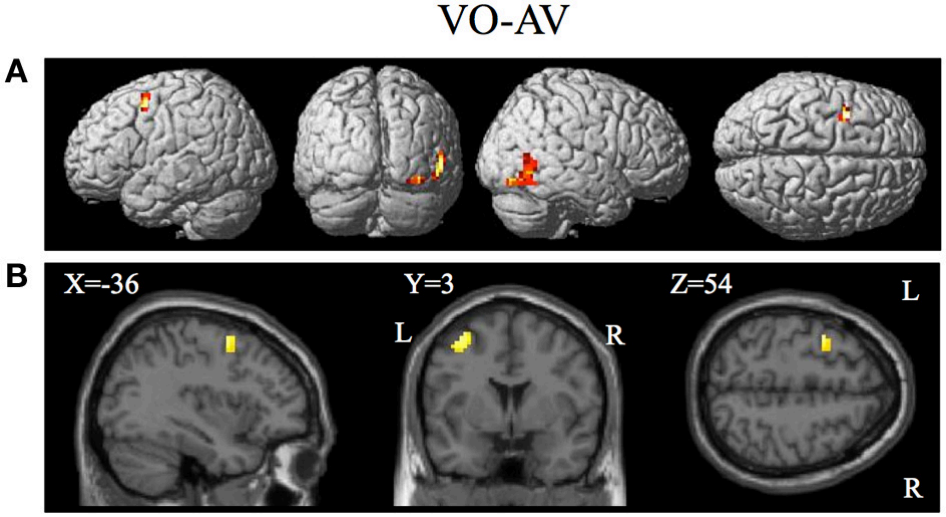
**Brain activity significantly active for the contrast of visual only VO relative to the combined AV conditions thresholded at *p* < 0.001 uncorrected**. Activity was present in the left PMvs/PMd and the left MT/V5 visual motion processing area. **(A)** Activity rendered on the surface of the left, back, right, and top of the brain. **(B)** Section through brain taken at MNI coordinate −36, 3, 54 shows activity that was present in the PMvs/PMd region. L, left side of brain; R, right side of brain.

The random effect results of the fMRI analyses of the contrasts of interest are given in Figures 3–8 and Tables 3–7. The brain activity rendered on the surface of the brain for the contrast of VO relative to baseline (still face plus button press) is given in Figure 3. Significant activity (pFDR < 0.05 corrected across entire volume; *T* = 4.38; see Table 3 for detailed results) was present in left PMvi/Broca's area, left PMvs/PMd, left and right middle temporal visual motion processing area (MT/V5). The results of the ROI analysis showed significant activity (*p* < 0.05 corrected; see Table 3) in the left PMvi/Brocas area (MNI coordinate: −48, 9, 12), the left PMvs/PMd (MNI coordinate: −39, 3, 54). Significant activity (pFDR < 0.05 corrected across entire volume; *T* = 3.28) for the combined AV conditions was present in left and right PMvi/Broca's area, left PMvs/PMd, left and right STG/S, left MT/V5, and right cerebellum lobule VIIb (see Figure 4 and Table 4). The results of the ROI analysis showed significant activity (*p* < 0.05 corrected; see Table 4) in the left PMvi/Broca's area (MNI coordinate: −51, 9, 9), the left PMvs/PMd (MNI coordinate: −48, 3, 42) and the right cerebellum lobule VIIb (MNI coordinate: 18, −72, −48). The conjunction of brain activity found to be active for both the combined AV conditions and the VO condition included the left PMvi/Broca's area, PMvs/PMd, and the left MT/V5 region (see Figure 5).

**Table 3:** **VO**.

**Brain region**	**MNI coordinates**	***T***
PMvi/Broca's	−48, 12, 9	7.97
BA6, 44		
PMvs/PMd	−39, 3, 54	4.70
BA6		
MT/V5	−51, −69, 0	7.33
	54, −66, −3	6.07

*Brain activity is thresholded using a false discovery rate FDR correction for multiple comparisons across the entire volume at pFDR < 0.05 for the Visual Only VO contrast. BA, Brodmann area; PMvi, Premotor ventral inferior; PMvs, Premotor ventral superior; PMd, Premotor dorsal; MT, Middle Temporal Gyrus; V5, Visual Area 5. Negative x MNI coordinates denote left hemisphere and positive x values denote right hemisphere activity*.

**Table 4:** **AV**.

**Brain region**	**MNI coordinates *x, y, z***	***T***
PMvi/Broca's	−51, 9, 9	8.37
BA6 and 44	48, 18, 18	4.61
PMvs/PMd	−48, 3, 42	4.61
BA6		
STG/S	−51, −33, 9	12.08
BA 22, 41, 42	66, −24, 0	12.93
MT/V5	−51, −63, 6	5.78
CerbLob VIIb	18, −72, −48	5.5

*Brain activity is thresholded using a false discovery rate FDR correction for multiple comparisons across the entire volume at pFDR < 0.05 for the combined (AV6, AV10, and AV14) audio visual AV contrast. BA, Brodmann area; PMvi, Premotor ventral inferior; PMvs, Premotor ventral superior; PMd, Premotor dorsal; STG/S, Superior temporal gyrus/sulcus; MT, Middle Temporal Gyrus; V5, Visual Area 5; CerbLob, Cerebellum Lobule. Negative x MNI coordinates denote left hemisphere and positive x values denote right hemisphere activity*.

**Table 5:** **(AV10-A10)-(AV6-A6)**.

**Brain region**	**MNI coordinates *x, y, z***	***T***
PMvi/Broca's	−54, 3, 15	5.2^*^
BA6, 44		
PreCG PostCG	−45, −18, 36	6.59
BA3, 4		
IPC/SMG BA40	−48, −36, 33	6.22
OccipLobe	33, −75, 6	4.91
CerbLob VIIb	21, −69, −45	4.38^*^
CerbLob IX	6, −51, −45	4.92
Brain stem	9, −30, −42	7.98^**^
	−6, −30, −42	5.75

*Brain activity is thresholded using p < 0.001 uncorrected, T = 3.73 for the multisensory enhancement contrast (AV10-A10)-(AV6-A6). BA, Brodmann area; PMvi, Premotor ventral inferior; PreCG, Pre-central gyrus; PostCG, Post-central gyrus; IPC, Inferior parietal cortex; SMG, Supramarginal Gyrus; OccipLobe, Occipital Lobe; CerbLob, Cerebellum Lobule. Negative x MNI coordinates denote left hemisphere and positive x values denote right hemisphere activity*.

* *Denotes significant activity using a small volume correction for multiple comparisons with a 10 mm search radius (see Methods for seed voxel coordinates for ROIs)*.

** *Denotes significant (pFDR < 0.05) correction for multiple comparisons over the entire volume*.

**Table 6:** **AV-VO**.

**Brain region**	**MNI coordinates**	***T***
STG/S	−45, −33, 6	13.2
BA22, 41, 42	57, −12, 3	11.23

*Brain activity is thresholded using a false discovery rate FDR correction for multiple comparisons across the entire volume at pFDR < 0.05 for the combined audio-visual relative to the visual only VO contrast. BA, Brodmann area; STG/S, Superior Temproal Gyrus/Sulcus. Negative x MNI coordinates denote left hemisphere and positive x values denote right hemisphere activity*.

**Table 7:** **VO-AV**.

**Brain region**	**MNI coordinates**	***T***
PMvs/PMd BA6	−39, 3, 54	4.79^*^
MT/V5	51, −66, −9	5.07
IOG V4	36, −78, −12	5.69

*Brain activity is thresholded using p < 0.001 uncorrected, T = 3.73 for the visual only relative to the combined audio-visual contrast. BA, Brodmann area; PMvs, Premotor ventral superior; MT, Middle Temporal Gyrus; V5, Visual Area 5; IOG, Inferior Occipital Gyrus; V4, Visual area 4. Negative x MNI coordinates denote left hemisphere and positive x values denote right hemisphere activity*.

* *Denotes significant activity using a small volume correction for multiple comparisons with a 10 mm search radius (see Methods for seed voxel coordinates for ROIs)*.

Brain regions involved with the audio-visual enhancement effect across different signal-to-noise ratios were investigated using the contrast of (AV10-A10)-(AV6-A6) as well as the contrast of (AV14-A14)-(AV10-A10). The (AV10-A10)-(AV6-A6) contrast shows the degree of audio-visual enhancement as reflected in the behavioral results (see Figure 2) was greater when the signal-to-noise ratio was −10 dB compared to −6 dB. Significant activity was only found in the brain stem using the FDR correction for multiple comparisons, therefore the results are shown using a threshold of *p* < 0.001 (*T* = 3.73) uncorrected (see Figure 6). Active brain regions included the left PMvi/Broca's area, left pre-central gyrus (PreCG) Post central gyrus (PostCG), left inferior parietal cortex/supramarginal gyrus (IPC/SMG), right occipital lobe, the right cerebellar lobule VIIb and IX, and the left and right brain stem (see Figure 6 and Table 5). The results of the ROI analysis showed significant activity (*p* < 0.05 corrected) in the left PMvi/Brocas area (MNI coordinate: −54, 3, 15), and the right cerebellum lobule VIIb (MNI coordinate: 21, −69, −45) (see Table 5). The behavioral results of the interaction of (AV14-A14)-(AV10-A10) did not show a significant multisensory enhancement effect (see Figure 2). Similarly, the results of the fMRI analysis for this contrast also did not reveal any significant activity (*p* > 0.05 uncorrected).

The contrasts investigating differences between the combined AV conditions and the VO condition are given in Figures 7–8 and Tables 6–7. The contrast of AV vs. VO revealed significant activity (pFDR < 0.05 corrected across entire volume, *T* = 3.48) in only the STG/S region also encompassing primary and secondary auditory cortex (see Figure 7 and Table 6). The results of the ROI analysis did not show any significant activity in the PMvi/Broca's, PMvs/PMd, or the cerebellum. The contrast of VO relative to the combined AV conditions did not show significant activity when using the FDR correction for multiple comparisons therefore the results are shown using a threshold of *p* < 0.001 uncorrected (*T* = 3.73; see Figure 8). Active brain regions include the left PMvs/PMd, and the right MT/V5, and the right inferior occipital gyrus (see Figure 8 and Table 7). The results of the ROI analysis (see Table 7) showed significant activity (*p* < 0.05 corrected) in the left PMvs/PMd (MNI coordinate: −39, 3, 54).

## Discussion

The purpose of this study was to determine if premotor regions, PMvi/Broca's and PMvs/PMd, as well as the cerebellum, demonstrate differential processing of multisensory (audio-visual) and unimodal (visual) speech gesture information. The primary finding was that the PMvi/Broca's area, the IPL, as well as the cerebellum showed properties of multisensory enhancement (see Figure 6 and Table 5), while the PMvs/PMd showed greater unimodal visual only processing (see Figure 8 and Table 7). It should be noted that activity in the speech motor areas, including the inferior frontal gyrus (including Broca's area) and a large portion of the PMC (including PMvi, PMvs, and PMd), was found for both the VO (see Figure 3 and Table 3) and the AV (see Figure 4 and Table 4) conditions. The activity in speech motor regions common to both of these conditions is shown by their conjunction in Figure 5.

It is often difficult to differentiate the brain networks that process the facial gestures that signal speech from the networks responsible for processing and integrating audio-visual speech stimuli because the intelligibility and task demands typically differ across conditions. Without controlling for these intelligibility differences, it is difficult to determine whether any increased brain activity reflects the processing of the visual and/or auditory features of speech, or is reflective of the level of intelligibility. As well, task difficulty can also confound the extent to which visual and audio-visual perception may show differential activity. This confound arises because activity in speech motor regions can be modulated by the degree of working memory and attention required for the speech task (Sato et al., 2009; Alho et al., 2012). We controlled for intelligibility and task demands in this experiment by utilizing a vowel identification task in which the presentation of visual information alone allowed perceptual performance that was equally high as the performance observed for the audio-visual condition. Indeed, there were no significant differences in behavioral performance for the conditions containing visual information (see Figure 1). These results suggest that the intelligibility did not differ between conditions and that the task demands as far as general working memory and attention are concerned were essentially the same.

It was hypothesized that the PMvi/Broca's area is a site in which multisensory information (auditory, visual, orosensory) and speech gesture motor information are integrated and show properties of multimodal enhancement (Wallace et al., 1992; Stein and Meredith, 1993; Callan et al., 2003). The brain imaging results (see Figure 6) of the (AV10-A10)-(AV6-A6) contrast showed activity related to the audio-visual enhancement effect (see Figure 2) when the signal-to-noise ratio of the audio signal was reduced. Of particular interest is activity denoting multisensory enhancement in the left hemisphere PMvi/Broca's, pre- and post-central gyrus, the IPC/SMG and the right cerebeullum lobule VIIb. These areas are all thought to be involved with forward and inverse internal models used to facilitate speech perception (Callan et al., 2004a; Rauschecker, 2011). Although these properties of multisensory enhancement were found in the PMvi/Broca's area it is not the case that this area was more strongly activated by the audio-visual stimuli than it was by the visual only stimuli in this study. The contrast of AV-V (see Figure 7 and Table 6) only shows activity in the STG/S and no significant activity even in the ROI analysis within PMvi/Broca's area. It is unclear why multisensory enhancement was not found in the STG/S, considering that multisensory enhancement has been observed in this area in other studies (Calvert et al., 2000; Callan et al., 2001, 2003, 2004b). It may not be too surprising that the brain imaging contrast between (AV14-A14)-(AV10-A10) did not show any significant brain activity given that the behavioral visual enhancement effect was also not significant (see Figure 2). One potential reason for the lack of an enhancement effect for this contrast may be that the audio signal was so low that there was not enough auditory information available to integrate with the visual information. This hypothesis is supported by the fact that the A14 condition did not significantly differ from chance performance, when corrections were made for multiple comparisons (see Figure 2 and Table 2).

We hypothesized that the PMvs/PMd region is involved with mapping unimodal aspects of sensory information onto speech articulatory gestures. The contrast of the visual only relative to the combined audio-visual conditions V-AV (see Figure 8, Table 7) showed activity in the left PMvs/PMd and the left MT/V5. The finding of differential activity in visual motion processing area MT/V5 is consistent with the assertion that a greater reliance on information in visual speech motion features is utilized when auditory information is not present. It is important to note that this activity is not a result of differences in task difficulty or intelligibility as these were the same between visual only V and audio-visual AV conditions.

The results of this study are consistent with the hypothesis that overlapping processes are carried out by PMvi/Broca's region and the PMvs/PMd region but that processing in these areas differ in the degree to which they process multisensory and unimodal stimuli. Within the context of an internal model based approach we propose that the nervous system relies to a greater degree on visual-articulatory based mappings when stimulus driven auditory-articulatory based mappings are not present. One could further conjecture that the PMvi/Broca's region may be more influenced by the ventral stream (what pathway) and the PMvs/PMd may be more influenced by the dorsal stream (where/how pathway). This is consistent with the model proposed by (Rauschecker and Scott, 2009; Rauschecker, 2011) in which the antero-ventral stream includes Broca's area PMv and the postero-dorsal stream includes the PMd. Multiple fiber tracts (Friederici, 2009) from superior temporal areas to IFG and PMC give support to the possibility of both antero-ventral and postero-dorsal streams including frontal speech regions. The inclusion of frontal speech areas in both the antero-ventral and postero-dorsal streams is in contrast to the model proposed by (Hickok and Poeppel, 2000, 2004, 2007) in which it is proposed that frontal speech areas (Broca's/PMvi; PMvs/PMd) are all thought to be within the postero-dorsal stream.

### Conflict of interest statement

The authors declare that the research was conducted in the absence of any commercial or financial relationships that could be construed as a potential conflict of interest.
